# 分散固相萃取-超高效液相色谱-串联质谱法测定农产品中乙拌磷及其代谢物

**DOI:** 10.3724/SP.J.1123.2021.04028

**Published:** 2022-02-08

**Authors:** Qiang SUN, Yubo LI, Guangyue WEN, Weimin WANG, Maofeng DONG, Hongxia TANG

**Affiliations:** 上海市农业科学院农药安全评价研究中心, 上海市农业科学院农产品质量标准与检测技术研究所, 上海 201106; Pesticide Safety Evaluation Research Center, Institute for Agro-Food Standards and Testing Technology, Shanghai Academy of Agricultural Sciences, Shanghai 201106, China; 上海市农业科学院农药安全评价研究中心, 上海市农业科学院农产品质量标准与检测技术研究所, 上海 201106; Pesticide Safety Evaluation Research Center, Institute for Agro-Food Standards and Testing Technology, Shanghai Academy of Agricultural Sciences, Shanghai 201106, China; 上海市农业科学院农药安全评价研究中心, 上海市农业科学院农产品质量标准与检测技术研究所, 上海 201106; Pesticide Safety Evaluation Research Center, Institute for Agro-Food Standards and Testing Technology, Shanghai Academy of Agricultural Sciences, Shanghai 201106, China; 上海市农业科学院农药安全评价研究中心, 上海市农业科学院农产品质量标准与检测技术研究所, 上海 201106; Pesticide Safety Evaluation Research Center, Institute for Agro-Food Standards and Testing Technology, Shanghai Academy of Agricultural Sciences, Shanghai 201106, China; 上海市农业科学院农药安全评价研究中心, 上海市农业科学院农产品质量标准与检测技术研究所, 上海 201106; Pesticide Safety Evaluation Research Center, Institute for Agro-Food Standards and Testing Technology, Shanghai Academy of Agricultural Sciences, Shanghai 201106, China; 上海市农业科学院农药安全评价研究中心, 上海市农业科学院农产品质量标准与检测技术研究所, 上海 201106; Pesticide Safety Evaluation Research Center, Institute for Agro-Food Standards and Testing Technology, Shanghai Academy of Agricultural Sciences, Shanghai 201106, China

**Keywords:** 超高效液相色谱-串联质谱, 分散固相萃取, 乙拌磷, 代谢物, 农产品, ultra high performance liquid chromatography-tandem mass spectrometry (UHPLC-MS/MS), dispersive solid phase extraction (d-SPE), disulfoton, metabolites, agricultural products

## Abstract

基于超高效液相色谱-串联质谱(UHPLC-MS/MS)建立定量分析乙拌磷及其5个代谢物(乙拌磷砜、乙拌磷亚砜、内吸磷-*S*、内吸磷-*S*-砜、内吸磷-*S*-亚砜)的方法,应用该方法分析其在农产品(豌豆、芦笋、小麦、咖啡豆和花生)中的残留量。对样品前处理及色谱条件进行优化,样品经过乙腈涡旋提取(小麦、咖啡豆和花生先加水润湿),经盐析分层,取上清液经50 mg C_18_、50 mg PSA和50 mg NH_2_混合分散固相萃取净化后,经Thermo Syncronis C_18_色谱柱(150 mm×2.1 mm, 5 μm)分离,柱温40 ℃,进样量2 μL,以水和乙腈为流动相梯度洗脱后,采用ESI源,在正离子扫描和多反应监测(MRM)模式下检测,外标法定量。结果表明,乙拌磷及其代谢物在2.0~200.0 μg/L范围内线性关系良好,相关系数(*R*^2^)≥0.9981。方法的检出限(LOD)为0.02~2.0 μg/kg,并以最小添加水平5 μg/kg为定量限(LOQ)。乙拌磷及其代谢物在豌豆、芦笋、小麦、咖啡豆和花生中5、100、1000 μg/kg 3个添加水平下的平均回收率为75.0%~110.0%,相对标准偏差(RSD)为0.7%~14.9%,方法的准确度和精密度符合农药残留测定。应用建立的方法对市售的40份小麦样品和40份花生样品进行检测,均低于方法的检出限。该方法具有操作简单、快速、灵敏、准确的特点,适用于谷物、油料、蔬菜等多种农产品中乙拌磷及其代谢物的残留检测。

## Abstract (en)

Disulfoton, an organophosphorus pesticide, is used to control cotton, beet, potato, and other seedling period aphids, leaf moths, underground pests, etc., with internal absorption, killing, gastric poisoning, and fumigation. Disulfoton is a highly toxic organophosphate pesticide, which can inhibit cholinesterase activity, resulting in neurophysiological disorders by inhalation, feeding, and transdermal absorption. Disulfoton is difficult to degrade in the environment, which leads to enrichment in organisms and interference with endocrine. This compound is harmful to the ecological environment and human health. To ensure the quality and safety of food, it is important to develop a detection method for disulfoton and its metabolites in agricultural products. A reliable method based on dispersive solid phase extraction (d-SPE) with ultra high performance liquid chromatography-tandem mass spectrometry (UHPLC-MS/MS) was developed for the determination of disulfoton and its metabolites (disulfoton sulfone, disulfoton sulfoxide, demeton-*S*, demeton-*S* sulfone, and demeton-*S* sulfoxide) in agricultural products (pea, asparagus, wheat, coffee bean, and peanut). The optimal extraction method was as follows: 5.0 g the samples were extracted with acetonitrile (wheat, coffee bean, and peanut presoaked in 5 mL water) in a 50 mL centrifuge tube, followed by 10 min vortex. Before 30 s vortex, 4 g NaCl was added. After 5 min centrifugation, 1.5 mL of the supernatant was cleaned up with 50 mg octadecylsilane bonded silica (C_18_), 50 mg primary secondary amine (PSA), and 50 mg aminopropyl (NH_2_) adsorbents. The analytes were separated on a Thermo Syncronis C_18_ column (150 mm×2.1 mm, 5 μm) with gradient elution using water and acetonitrile at a column temperature of 40 ℃. The injection volume was 2 μL. Disulfoton and its metabolites were analyzed in multiple reaction monitoring (MRM) mode with positive electrospray ionization (ESI^+^) for the selective quantification. Qualitative and quantitative analyses were accorded to the retention times and characteristic ion pairs with one parent ion and two fragment ions. Quantitative analysis was performed by an external standard method using matrix-matched calibration curves. All the parameters that affected the extraction efficiencies were optimized. C_18_, PSA, and NH_2_ gave good recoveries of 87.9%-109.0%. Other adsorbents, multiwalled carbon nanotubes (MWCNTs), hydroxylated multiwalled carbon nanotubes (OH-MWCNTs), carboxylated multiwalled carbon nanotubes (COOH-MWCNTs), octylsilane bonded silica (C_8_), strong cation exchange (SCX) and neutral alumina (*N*-Al_2_O_3_), led to recoveries below 56.2%. The combination of adsorbents was also considered. Seven different combinations of 50 mg C_18_, 50 mg PSA, and 50 mg NH_2_ were chosen for the optimization experiments. There were no obvious differences in these combinations, and the target analytes recoveries ranged from 81.0% to 109.3% with relative standard deviations (RSDs) between 0.6% and 12.5%. The matrix effect could affect the extraction efficiency. The adsorbents of 50 mg C_18_, 50 mg PSA, and 50 mg NH_2_ showed weaker matrix effects as compared with other combinations of adsorbents in the instrument. The results for the matrix effect showed that peanuts and asparagus exceeded 20%, requiring matrix-matched calibration curves. Under the optimized conditions, disulfoton and its metabolites showed good linearities (*R*^2^≥0.9981) in the range of 2.0-200.0 μg/L. The average spiked recoveries of disulfoton and its metabolites in peas, asparagus, wheat, peanuts, and coffee beans ranged from 75.0% to 110.0%, with RSDs of 0.7% to 14.9%. The limits of detection (LODs) were between 0.02 and 2.0 μg/kg, and the limits of quantification (LOQs) were 5.0 μg/kg. The method was applied for the detection of 80 commercial productions, and neither disulfoton nor its metabolites were found. The proposed method is rapid, accurate, highly selective, and sensitive, and it is suitable for the simultaneous determination of disulfoton and its metabolites in grain, oil crops, vegetables, and other matrices.

乙拌磷是一种有机磷杀虫剂,其化学名称为*O*,*O*-二乙基-*S*-2-乙硫基乙基二硫代酸酯,可用于防治棉花、甜菜、马铃薯等苗期蚜虫、叶螨及地下害虫等,具有内吸、触杀、胃毒及熏蒸作用。乙拌磷纯品是一种具有恶臭味的无色油状物,易溶于正己烷、二氯甲烷、异丙醇和甲苯,难溶于水,在碱性条件下会分解失效。乙拌磷是一种高毒类有机磷农药,人体吸入、食入和经皮吸收后会抑制胆碱酯酶活性,造成神经功能紊乱,在环境中难降解,在生物体内富集,干扰生物体的内分泌机制,对生态环境和人体健康造成巨大危害^[1,2,3]^。乙拌磷在动植物体内均快速吸收和代谢,主要代谢产物是乙拌磷砜和乙拌磷亚砜,以及他们相应的氧类似物及二乙基硫代磷酸酯^[4]^,而且代谢物的降解速度均比乙拌磷缓慢^[3]^。我国规定剧毒、高毒农药不得用于防治卫生害虫,不得用于蔬菜、瓜果、茶叶、菌类、中草药材的生产^[5]^。因此,为确保食品质量安全,开发农产品中乙拌磷及其代谢物的检测方法至关重要。

目前《食品安全国家标准 食品中农药最大残留限量》^[6]^和农药残留联席会议(JMPR)^[7]^将乙拌磷的残留定义为乙拌磷、硫醇式-内吸磷以及它们的亚砜化物和砜化物含量之和,以乙拌磷表示。文献报道测定植物源食品中乙拌磷的方法包括气相色谱法^[8]^和气相色谱-串联质谱法^[9]^。有报道采用液相色谱-串联质谱法^[2]^检测人体血液和尿液中乙拌磷及其5个代谢物残留量;气相色谱法^[3]^测定种植咖啡土壤中乙拌磷的降解和迁移,检测乙拌磷及其5个代谢物;双气相色谱-双脉冲火焰光度法^[10]^检测动物性食品中乙拌磷、乙拌磷亚砜、乙拌磷砜和内吸磷-*S*;固相萃取柱净化-液相色谱-串联质谱法^[11]^检测当归中乙拌磷、乙拌磷砜和乙拌磷亚砜。但是鲜有检测植物源性食品中乙拌磷及其5个代谢物的相关报道。因此,为保证植物源农产品质量安全,本研究拟采用UHPLC-MS/MS建立灵敏度高、简便快速的定量分析乙拌磷及其代谢物的残留方法。

目前农药残留前处理方法主要有固相萃取法、分散液液萃取法和分散固相萃取法等^[12,13,14]^。固相萃取法操作步骤复杂且时间长,消耗溶剂量大,商品化固相萃取小柱种类多,由于化合物结构特点不同,难以选择一种合适的固相萃取小柱;分散液液萃取法需要消耗大量的有机溶剂且当化合物存在干扰时,无有效的解决措施;分散固相萃取法结合了液液萃取和固相萃取的原理,利用少量吸附剂加入到提取液吸附杂质,起到净化效果。对比固相萃取法和分散液液萃取法,分散固相萃取操作步骤简单、快速,净化过程中不用额外的有机试剂。

本研究根据目标化合物的性质,采用分散固相萃取法,结合国际食品法典委员会(CAC)和我国关于乙拌磷的残留限量要求,建立了5种典型性农产品(豌豆、芦笋、小麦、咖啡豆和花生)中乙拌磷及其代谢物农药残留的UHPLC-MS/MS测定方法。本方法具有操作简单便捷,适用于谷物、油料、蔬菜等多种基质中乙拌磷及其代谢物的定性和定量分析。

## 1 实验部分

### 1.1 仪器、试剂与材料

LC-30A超高效液相色谱仪、8060三重四极杆质谱仪(配电喷雾电离(ESI)源)(日本Shimadzu公司); MX-F涡动混合器(中国Dragonlab公司); 5415D离心机(德国Eppendorf公司)。

乙拌磷(99.4 mg/L)、乙拌磷砜(1000.1 mg/L)、乙拌磷亚砜(1000 mg/L)、内吸磷-*S*(1000.2 mg/L)、内吸磷-*S*-亚砜(纯度96%)购自北京振翔科技有限公司;内吸磷-*S*-砜(100 mg/L)购自天津阿尔塔科技有限公司。乙腈和甲醇(色谱纯)购自美国Merck公司;甲酸和乙酸铵(色谱纯)购自上海安谱实验科技股份有限公司;氯化钠和无水硫酸镁(分析纯)购自上海化学试剂公司。多壁碳纳米管(MWCNT)、羟基化多壁碳纳米管(OH-MWCNTs)、羧基化多壁碳纳米管(COOH-MWCNTs)购自江苏先丰纳米材料科技有限公司;辛烷基硅烷键合硅胶(C_8_)购自上海安谱实验科技股份有限公司;氨丙基粉(NH_2_)、中性氧化铝(*N*-Al_2_O_3_)、硅胶键合苯基磺酸强阳离子交换吸附剂(SCX)购自安捷伦科技(中国)有限公司;乙二胺-*N*-丙基硅烷(PSA)、十八烷基键合硅胶(C_18_)和石墨化炭黑(GCB)购自日本Shimadzu公司。

样品:在广西、河南、南京和上海分别采集10批次小麦,总计40个样品;在河南、上海、浙江和山东分别采集10批次花生,总计40个样品。咖啡豆(鲜)等其他样品均购于网络。

### 1.2 标准溶液配制

准确称取内吸磷-*S*-亚砜10.0 mg,用甲醇溶解并定容至10 mL,得到960 mg/L的内吸磷-*S*-亚砜标准储备液。

分别量取1.006、0.100、0.100、0.100、0.104和1.000 mL的乙拌磷、乙拌磷砜、乙拌磷亚砜、内吸磷-*S*、内吸磷-*S*-亚砜和内吸磷-*S*-砜标准储备液,用甲醇定容至10 mL,得到混合标准中间液。将上述混合标准中间液用空白基质提取液稀释成质量浓度为2、10、50、100、200 μg/L的系列混合标准溶液。

所有标准溶液均保存于-(18±2) ℃条件下。

### 1.3 样品前处理

将豌豆或芦笋样品切碎、匀浆,将小麦、咖啡豆或花生样品粉碎后充分混匀。

称取试样5.0 g(精确至0.001 g),置于50 mL离心管中,加入10 mL乙腈(其中小麦、咖啡豆或花生样品先加入5 mL水润湿),涡旋提取10 min,加入4 g氯化钠,再次涡旋30 s,再以4000 r/min离心5 min,准确移取1.5 mL提取上清液至盛有50 mg C_18_、50 mg PSA和50 mg NH_2_的2 mL净化离心管中,涡旋1 min后,以10000 r/min离心2 min,取上清液过0.22 μm有机微孔滤膜后上机测定。

### 1.4 仪器条件

1.4.1 色谱条件

Thermo Syncronis C_18_色谱柱(150 mm×2.1 mm, 5 μm);柱温40 ℃;流动相A为乙腈,B为水;流速0.50 mL/min。梯度洗脱程序:0~1.0 min, 10%A; 1.0~6.0 min, 10%A~90%A; 6.0~7.0 min, 90%A; 7.0~7.5 min, 90%A~10%A; 7.5~10 min, 10%A。进样量2 μL。

1.4.2 质谱条件

ESI^+^检测方式;多反应监测(MRM)模式;离子源接口电压:3.5 kV;脱除溶剂管(DL管)温度:250 ℃;加热模块温度:400 ℃;雾化气流速:3.0 L/min (N_2_,纯度99.999%);干燥气流速:15 L/min(N_2_,纯度99.999%)。乙拌磷及其代谢物质谱参数见表1。

**表 1:** 乙拌磷及其代谢物的质谱参数

Compound	Retention time/min	Precursor ion (m/z)	Product ion (m/z)	Collision energy/V
Disulfoton	5.59	275.00	61.05^*^	-33
			89.15	-22
Disulfoton sulfone	5.32	307.05	97.00^*^	-33
			125.10	-18
Disulfoton sulfoxide	4.87	291.00	213.05^*^	-10
			185.00	-14
Demeton-S	5.27	259.05	89.05^*^	-22
			61.00	-34
Demeton-S sulfone	4.02	291.05	263.00^*^	-13
			235.00	-16
Demeton-S sulfoxide	3.58	275.00	141.05^*^	-21
			197.05	-12

* Quantitative ion.

## 2 结果与讨论

### 2.1 质谱条件优化

首先,对乙拌磷及其代谢物的质谱条件进行优化,将目标化合物的标准溶液在ESI^+^和ESI^-^两种模式下进行扫描。结果显示,乙拌磷及其代谢物在ESI^+^模式下得到[M+H]^+^准分子离子峰,质谱信号响应比ESI^-^模式强,因此选择正离子模式开展后续优化。选择乙拌磷及其代谢物的特征准分子离子峰为前体离子进行二级质谱分析,每个目标化合物选取2对响应最强的特征离子作为定性离子,以响应值最大的碎片离子为定量离子,自动优化质谱参数,乙拌磷及其代谢物的最佳质谱条件见表1。

### 2.2 色谱条件优化

2.2.1 色谱柱选择

实验对比了Agilent EC-C_18_ (100 mm×3.0 mm, 2.7 μm)、Thermo Syncronis C_18_ (150 mm×2.1 mm, 5 μm)、Waters Acquity C_18_ (50 mm×2.1 mm, 1.7 μm)、Shimadzu InertSustainSwift C_18_ (50 mm×2.1 mm, 3 μm)4种液相色谱柱对乙拌磷及其代谢物的分离效果。如图1所示,所有化合物在4种色谱柱上均能实现有效保留及分离,但采用Agilent EC-C_18_和Thermo Syncronis C_18_色谱柱时峰形对称性好,半峰宽窄,且出峰时间相同,但采用Thermo Syncronis C_18_色谱柱时峰面积明显高于Agilent EC-C_18_色谱柱。因此最终选择Thermo Syncronis C_18_色谱柱进行分离。

**图1:**
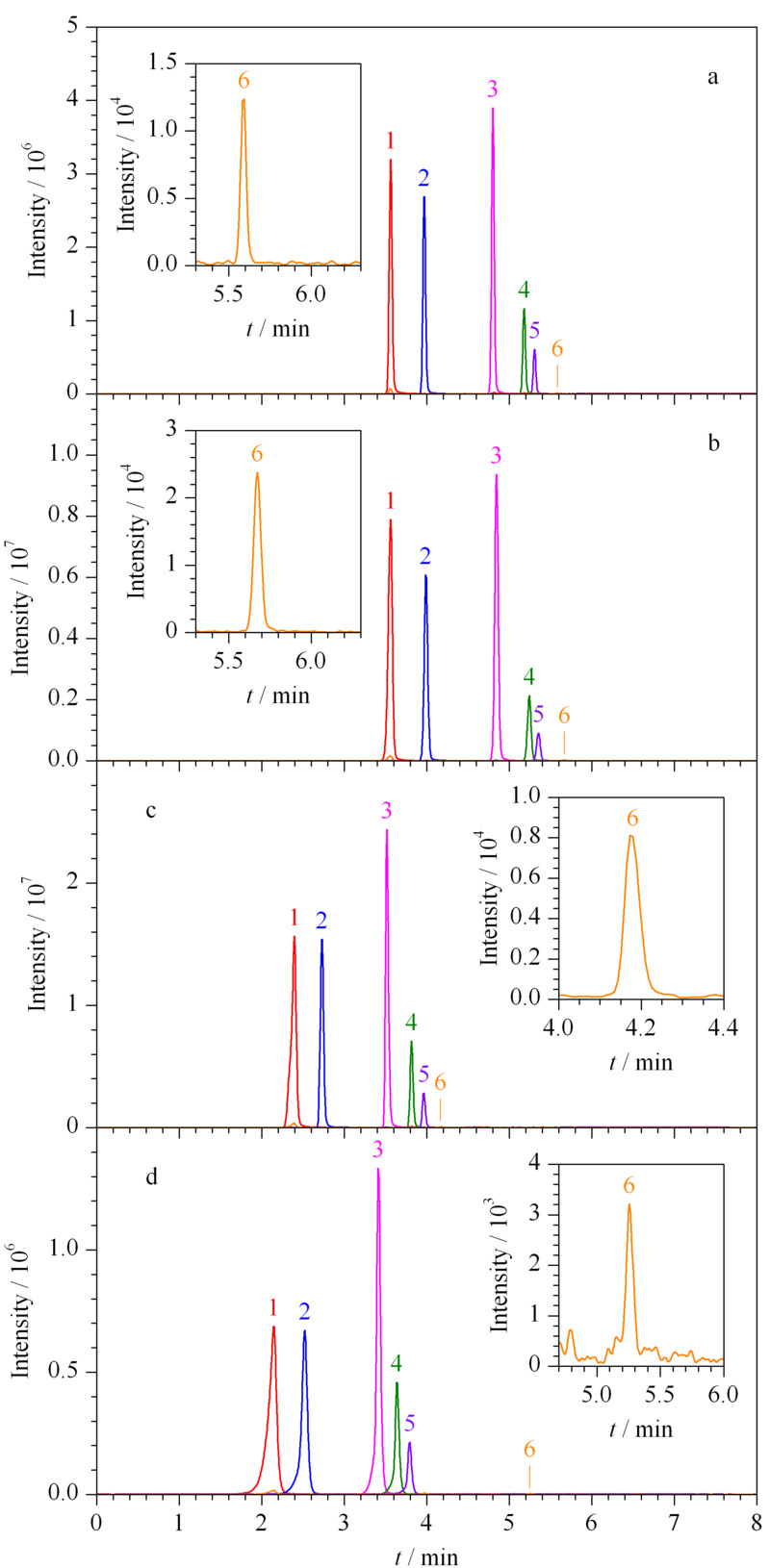
采用不同色谱柱时乙拌磷及其代谢物(0.05 mg/L)的总离子流色谱图

2.2.2 流动相选择

对于液相色谱-串联质谱法,流动相条件是影响目标化合物分离度和响应的一个重要方面。在水相中添加甲酸铵或甲酸等试剂是改善色谱峰形、提高仪器响应值和离子化效率的常用手段,通常采用酸性流动相有利于在正离子模式下进行质谱检测,而甲酸是最常用的试剂之一。以乙拌磷及其代谢物的质量浓度为0.05 mg/L为实验条件,首先比较甲醇-水(见图2a)和乙腈-水(见图2b)两种流动相对6种化合物分离度的影响。结果表明,采用乙腈-水作为流动相,乙拌磷及其代谢物的分离度明显优于甲醇-水。其次比较了乙腈-水和乙腈-0.05%甲酸水(见图2c)两种流动相对6种化合物响应的影响。如图显示,6种化合物在乙腈-水流动相中,峰高更高。因此最终采用乙腈-水作为流动相。

**图2:**
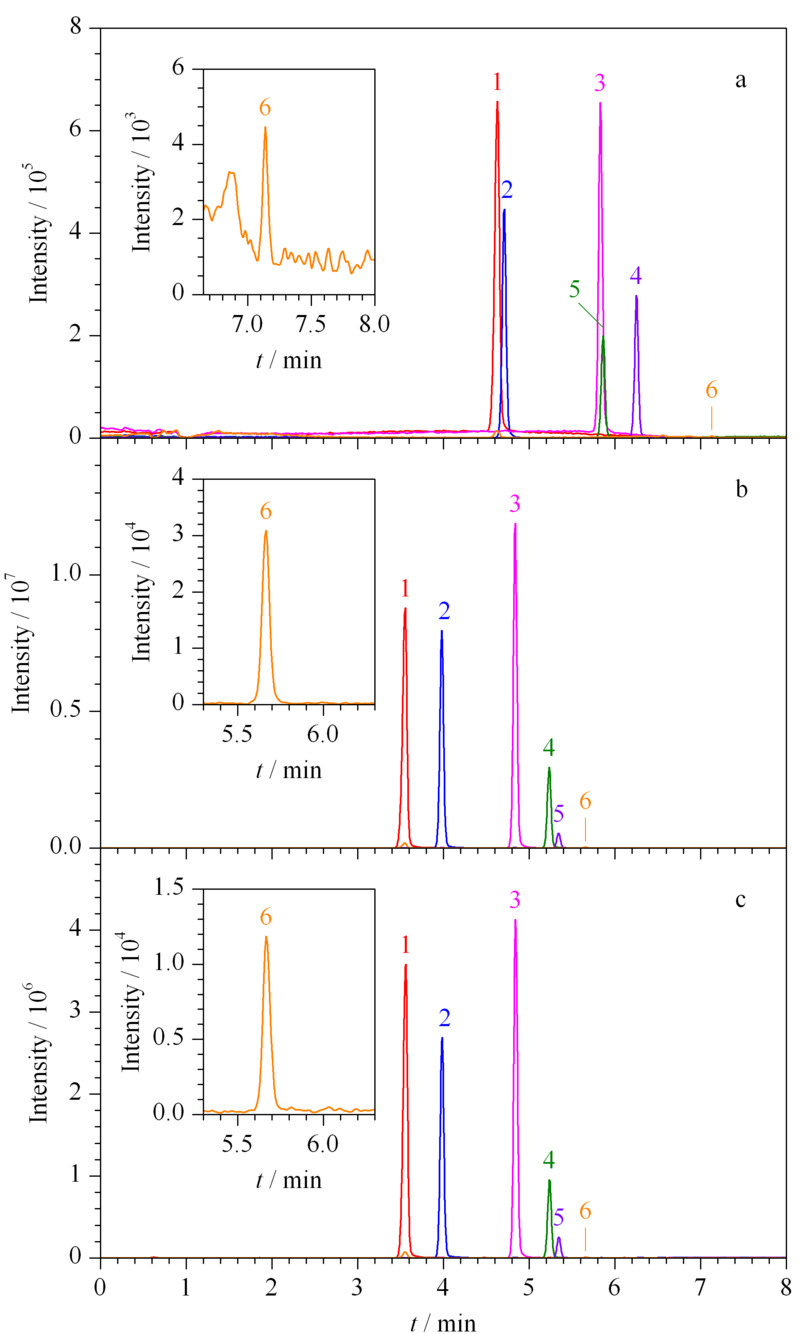
采用不同流动相时乙拌磷及其代谢物(0.05 mg/L)的总离子流色谱图

### 2.3 吸附剂种类的优化

实验选择MWCNTs、OH-MWCNTs、COOH-MWCNTs、C_8_、C_18_、NH_2_、SCX、*N*-Al_2_O_3_、GCB和PSA 10种常见的分散固相萃取吸附材料,考察了其对目标化合物的吸附情况。分别称取10 mg MWCNTs、OH-MWCNTs、COOH-MWCNTs和50 mg C_8_、C_18_、NH_2_、SCX、*N*-Al_2_O_3_、GCB和PSA,对乙拌磷及其代谢物(0.05 mg/L)在乙腈中吸附情况进行考察。如图3a结果所示,MWCNTs、OH-MWCNTs、COOH-MWCNTs、C_8_、SCX或*N*-Al_2_O_3_净化时内吸磷-*S*亚砜的回收率都低于56.2%; GCB净化时,乙拌磷的回收率偏低(71.5%);其余3种吸附剂(C_18_、NH_2_或PSA)净化时,乙拌磷及其代谢物的回收率为87.9%~109.0%,均能满足检测方法的要求。

**图3:**
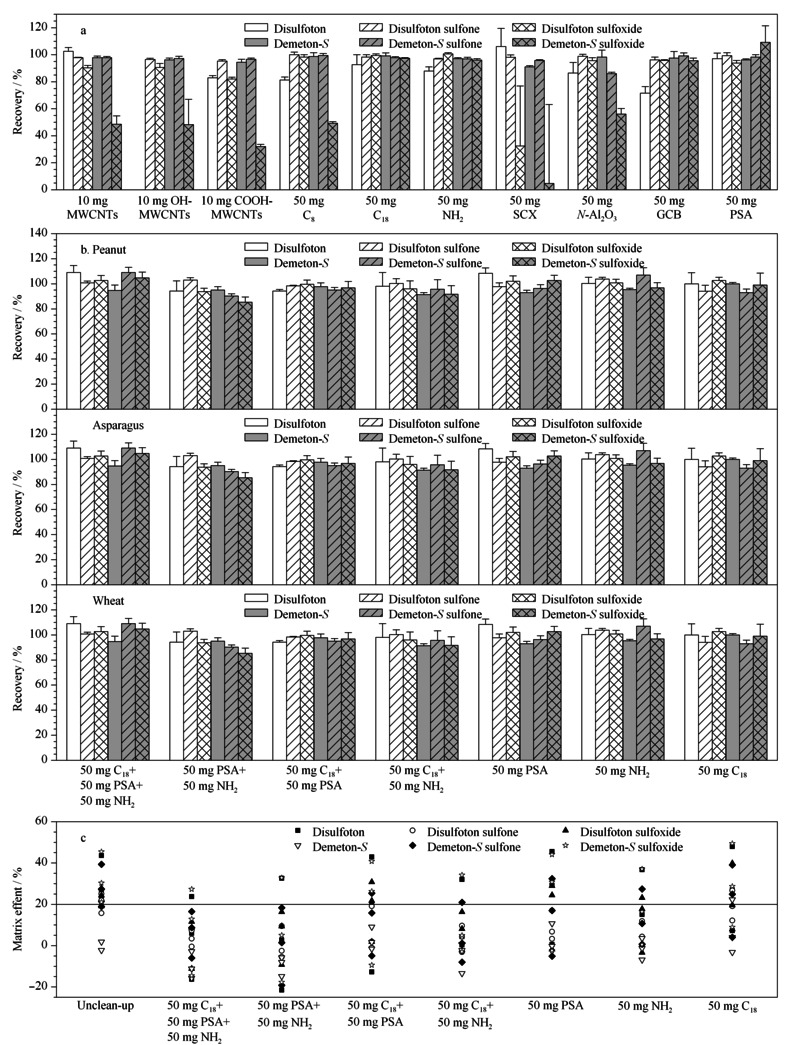
不同吸附剂对乙拌磷及其代谢物(a、b)回收率和(c)基质效应的影响(*n*=5)

### 2.4 吸附剂用量的优化

C_18_主要吸附脂类和甾醇等非极性杂质^[15]^, NH_2_主要吸附碳水化合物和部分脂肪^[16]^, PSA主要吸附极性有机酸、脂肪酸、极性色素和糖^[15,17]^。鉴于本方法拟适用于谷物、油料和蔬菜等多种基质,选择了具有代表性的花生(油脂类)、芦笋(高含水蔬菜类)、小麦(谷物类)3种基质进一步开展C_18_、NH_2_和PSA不同组合的回收率和基质效应的评价。

结合上述实验结果,以高含油量作物(花生)、高含水量作物(芦笋)和高淀粉含量作物(小麦)为基质,分别选定不同吸附材料(见图3b)处理净化后的提取液配制乙拌磷及其代谢物的标准溶液(0.05 mg/L),进行添加回收评价。如图3b所示,7种吸附剂组合方式下,乙拌磷及其代谢物在花生、芦笋和小麦中的回收率均无明显影响,目标化合物的回收率为81.0%~109.3%,相对标准偏差(RSD)为0.6%~12.5%,均满足分析方法要求。

### 2.5 基质效应的探究

国际纯粹与应用化学联合会(IUPAC)定义基质效应是指样品中除分析物以外的其他成分对待测物测定值的综合影响。基质效应一般受分析仪器、样品基质、前处理方式等因素的影响^[12,18]^。样品基质中存在对待测成分有干扰的化合物,在UHPLC-MS/MS分析中,离子化过程时,干扰成分同待测成分离子化竞争,从而引起待测成分信号抑制或增强,对方法的灵敏度、精密度和准确度造成影响。

根据文献^[15,19]^报道, ME=(基质中分析物的峰面积/纯溶剂中分析物的峰面积-1)×100%。当ME>0,为基质增强效应;当ME=0,无基质效应;当ME<0,为基质抑制效应。一般认为当|ME|≤20%,认为该基质没有基质效应;若在20%~50%之间,表明有中等基质效应;当大于50%,对目标化合物有较强的基质效应。实验比较了溶剂标准溶液(0.05 mg/L)、7种吸附剂组合方式净化后基质匹配标准溶液和未净化的基质匹配标准溶液。

由图3c可知,乙拌磷及其代谢物在7种预定方式净化后,基质效应绝对值均呈明显下降,但是采用50 mg PSA、50 mg NH_2_和50 mg C_18_混合时净化效果最佳。在3种吸附材料净化条件下,仍然有部分|ME|值超过20%(花生基质中的乙拌磷和芦笋基质中的硫醇式-内吸磷亚砜)。因此,为了确保分析方法的准确性,尤其在油料和蔬菜基质中,仍然需要通过配制基质标准溶液进行定量分析。

### 2.6 线性关系、相关系数和检出限

根据1.2节配制基质混合标准溶液,分别进样2 μL,在优化后的色谱及质谱条件下进行测定,以各分析物的质量浓度(*x*, μg/L)为横坐标,以其相应的峰面积(*y*)为纵坐标,得到各分析物的线性方程。结果显示,乙拌磷及其代谢物在2.0~200.0 μg/L线性范围内呈现出良好的线性关系,相关系数(*R*^2^)≥0.9981。乙拌磷及其代谢物的检出限(LOD)为3倍信噪比所对应的含量,为0.02~2.0 μg/kg(见表2)。根据欧盟文件SANTE/11813/2017规定,当最小添加水平回收率满足70%~120%及相对标准偏差≤20%时,定量限(LOQ)可以为最小添加水平。本实验的最小添加水平为5 μg/kg,乙拌磷及其代谢物的回收率≥75.0%, RSD≤14.9%,因此乙拌磷及其代谢物的LOQ为5 μg/kg。

**表 2:** 乙拌磷及其代谢物的回归方程、相关系数、检出限、回收率和相对标准偏差(*n*=5)

Compound	Matrix	Regression equation	R^2^	LOD/ (μg/kg)	Recoveries (RSDs) (n=5)/%		
					5 μg/kg	100 μg/kg	1000 μg/kg
Disulfoton	pea	y=9.29×10^2^x-1.55×10^3^	0.9981	2.0	93.6 (13.7)	90.4 (10.7)	89.0 (9.5)
	asparagus	y=4.60×10^2^x-6.20×10^2^	0.9988	2.0	93.8 (14.4)	97.8 (7.9)	96.0 (3.8)
	wheat	y=2.49×10^2^x+4.66×10^2^	0.9996	2.0	79.6 (10.8)	84.0 (12.1)	83.4 (9.1)
	peanut	y=2.64×10^2^x-3.91×10^2^	0.9983	2.0	99.2 (9.3)	95.0 (9.2)	104.2 (6.3)
	coffee bean	y=7.16×10^2^x+4.95×10^2^	0.9995	2.0	81.6 (9.1)	76.4 (7.3)	103.2 (6.4)
Disulfoton sulfone	pea	y=2.94×10^4^x-4.97×10^4^	0.9986	0.1	79.4 (4.8)	106.6 (6.9)	75.0 (3.3)
	asparagus	y=7.04×10^3^x-8.24×10^3^	0.9991	0.2	101.0 (7.8)	102.6 (5.5)	80.2 (2.8)
	wheat	y=4.15×10^3^x-3.91×10^3^	0.9994	0.5	79.2 (9.8)	81.2 (10.8)	79.8 (5.3)
	peanut	y=1.00×10^4^x+1.47×10^4^	0.9993	0.1	110.0 (6.6)	92.0 (7.7)	93.4 (4.3)
	coffee bean	y=1.32×10^4^x+1.59×10^4^	0.9998	0.09	80.4 (9.9)	103.0 (3.0)	103.2 (3.8)
Disulfoton sulfoxide	pea	y=1.61×10^5^x-2.25×10^5^	0.9999	0.02	104.4 (6.0)	99.0 (5.8)	83.6 (9.8)
	asparagus	y=3.62×10^4^x-3.58×10^4^	0.9993	0.05	90.6 (4.2)	106.4 (4.6)	99.4 (5.2)
	wheat	y=1.96×10^4^x+2.65×10^4^	0.9996	0.1	80.2 (14.9)	82.4 (6.5)	84.4 (8.7)
	peanut	y=7.27×10^4^x-1.03×10^5^	0.9997	0.04	93.8 (3.8)	89.0 (3.2)	92.8 (2.8)
	coffee bean	y=9.16×10^4^x-7.05×10^4^	0.9997	0.02	87.8 (10.3)	101.0 (0.7)	101.8 (0.8)
Demeton-S	pea	y=5.04×10^4^x-5.38×10^4^	0.9997	0.04	88.8 (10.2)	86.4 (11.2)	105.0 (6.1)
	asparagus	y=1.96×10^4^x+1.56×10^3^	0.9999	0.1	93.4 (1.4)	100.6 (5.5)	105.0 (1.8)
	wheat	y=1.32×10^4^x+2.51×10^4^	0.9984	0.2	94.2 (11.4)	80.6 (8.4)	77.0 (4.9)
	peanut	y=4.03×10^4^x+1.39×10^4^	0.9997	0.04	88.6 (4.3)	90.2 (1.8)	99.2 (3.2)
	coffee bean	y=4.36×10^4^x+8.87×10^4^	0.9994	0.03	88.0 (2.8)	102.4 (1.5)	103.4 (3.3)
Demeton-S sulfone	pea	y=8.70×10^4^x-1.92×10^5^	0.9995	0.02	79.6 (6.6)	105.8 (7.3)	79.6 (6.7)
	asparagus	y=1.75×10^4^x-2.63×10^4^	0.9993	0.1	88.6 (9.3)	107.6 (7.4)	86.2 (3.1)
	wheat	y=7.86×10^3^x-1.19×10^4^	0.9994	0.3	83.2 (14.7)	76.2 (5.8)	79.8 (9.1)
	peanut	y=4.12×10^4^x-6.54×10^4^	0.9988	0.04	105.8 (5.2)	85.6 (6.0)	87.6 (2.5)
	coffee bean	y=5.16×10^4^x+8.34×10^3^	0.9999	0.04	96.0 (11.7)	96.2 (0.9)	93.0 (1.7)
Demeton-S sulfoxide	pea	y=7.47×10^4^x-5.29×10^4^	0.9999	0.03	96.6 (3.0)	104.0 (6.8)	94.2 (8.5)
	asparagus	y=2.97×10^4^x+5.01×10^4^	0.9997	0.06	95.4 (5.2)	105.8 (7.9)	105.8 (3.6)
	wheat	y=8.12×10^3^x-1.08×10^4^	0.9991	0.3	81.4 (14.0)	86.2 (3.0)	86.0 (6.8)
	peanut	y=5.78×10^4^x-6.57×10^4^	0.9995	0.03	84.8 (10.9)	87.0 (13.0)	87.0 (1.4)
	coffee bean	y=8.99×10^4^x+1.44×10^5^	0.9995	0.02	103.0 (7.3)	90.8 (1.4)	93.2 (3.3)

*y*: peak area of quantitative ion; *x*: mass concentration, μg/L.

### 2.7 回收率和精密度

选择不含待测物的样品(豌豆、芦笋、小麦、咖啡豆和花生)进行添加回收试验,添加水平分别为5、100和1000 μg/kg,每个样品基质做5次平行试验,最高添加水平用空白基质稀释5倍后检测。结果如表2所示,乙拌磷及其代谢物的回收率为75.0%~110.0%, RSD为0.7%~14.9%。符合《农作物中农药残留试验准则》的要求^[20]^。

### 2.8 实际样品的测定

选取各地市售的40份小麦、40份花生样品,采用所建立的方法,按照最优实验条件进行乙拌磷及其代谢物残留的检测。检测结果显示,所有样品均未检出乙拌磷及其代谢物。

## 3 结论

本文建立了分散固相萃取-超高效液相色谱-串联质谱测定乙拌磷及其代谢物在不同农产品中的检测方法,该方法有较好的检测灵敏度和准确度。该方法操作简单,灵敏度高,回收率高,准确度好,检出限低,满足定性和定量要求,适用于高含水类、高淀粉含量类和高含油量类作物中乙拌磷及其代谢物的检测,可为乙拌磷在高淀粉含量类和高含油量类作物的风险监控提供有效的技术支持。
